# Single-Port Subcostal Robot-Assisted Minimally Invasive Esophagectomy—How to Do It?

**DOI:** 10.1055/a-2587-6701

**Published:** 2025-05-14

**Authors:** Edin Hadzijusufovic, Vladimir J. Lozanovski, Luca Bellaio, Evangelos Tagkalos, Eren Uzun, Eva-Verena Griemert, Hauke Lang, Peter P. Grimminger

**Affiliations:** 1 Department of General, Visceral and Transplantation Surgery, University Medical Center of the Johannes Gutenberg University Mainz, Mainz, Germany; 2 Department of Anesthesia, University Medical Center of the Johannes Gutenberg University Mainz, Mainz, Germany

**Keywords:** minimally invasive surgery, robotic, esophageal surgery

## Abstract

Minimally invasive robot-assisted esophagectomies have proven superior to traditional open surgery. While transhiatal and transthoracic approaches are common, subcostal access remains less frequent in minimally invasive esophageal surgery. Recent advancements in robotic systems, such as the da Vinci Single-Port (SP), now facilitate precise subcostal access. This innovation holds potential to reduce postoperative pain, enhance patient mobility, and broaden surgical options for patients with multiple health conditions. The Single-Port Subcostal Robot-Assisted Minimal Invasive Esophagectomy (SP SC RAMIE) utilizes an SP and laparoscopic approach, enabling effective mediastinal dissection and esophageal mobilization with radical lymphadenectomy. This novel technique shows promise, especially for frail patients with multiple comorbidities who stand to benefit greatly from expedited recovery pathways. Nonetheless, further exploration is necessary to fully assess its clinical effectiveness and reproducibility.

## Introduction

Minimally invasive esophagectomies have shown significant advantages over traditional open surgery. 1 2 3 Typically, robot-assisted esophageal surgeries use either transhiatal or transthoracic approaches. However, advancements in robotic systems, such as the da Vinci Single-Port (SP; Intuitive Surgical Inc., Sunnyvale, CA), have enabled more precise access to the esophagus through a small SP incision. This incision is used during the thoracic phase to prepare the esophagus and can be used for specimen removal and reconstruction. This innovative approach is expected to reduce pain and the necessity for painkillers while enhancing patient mobility, thereby lowering the risks of thromboembolism and pulmonary complications like pneumonia. The Single-Port Subcostal Robot-Assisted Minimally Invasive Esophagectomy (SP SC RAMIE) method combines laparoscopy with SP access subcostal thoracoscopy for mediastinal esophageal dissection. To date, SP SC RAMIE has been performed in a cadaveric study. 4 In this paper, we describe this novel subcostal robotic approach using the da Vinci SP system.

## Technique

The SP SC RAMIE procedure was frequently performed at the University Hospital Mainz, in the Department of General, Visceral and Transplantation Surgery, in May 2024.

### System and Instruments

The da Vinci SP System (Intuitive Surgical, Sunnyvale, CA) and the corresponding instrumentation are used.

### Surgical Procedure

#### Abdominal Phase

This part of the procedure has been previously described. 5

#### Subcostal Single-Port Phase

**Fig. 1:**
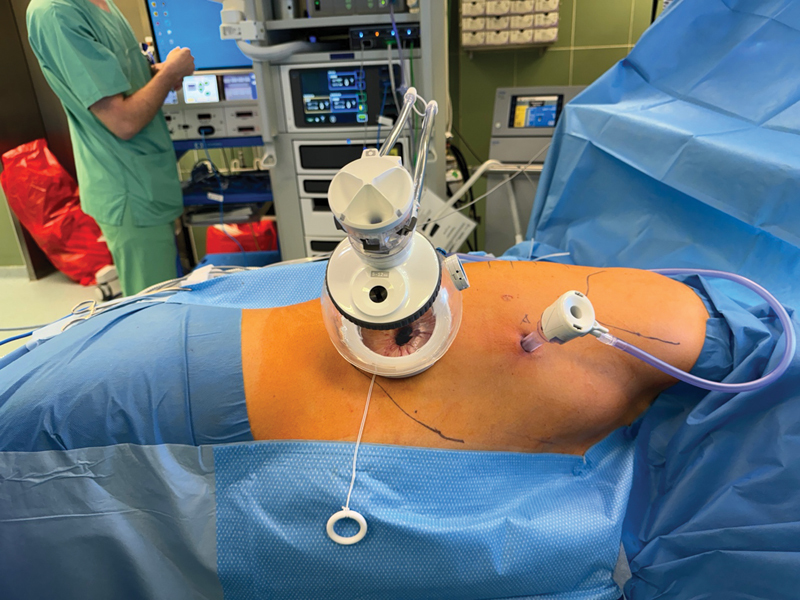
Large incision (2.7–7 cm) SP access port kit is inserted, and capnopneumothorax is induced. SP, single-port.

**Fig. 2:**
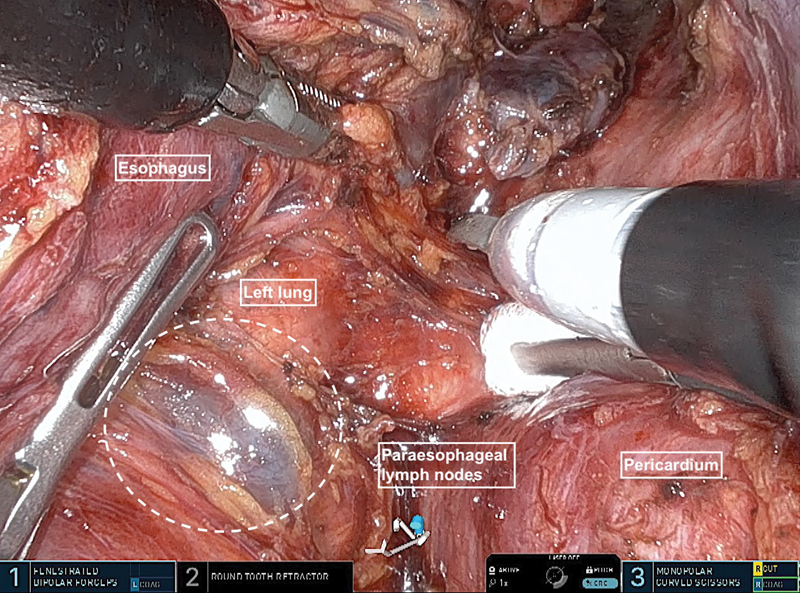
The esophagus is tunneled underneath, and the lymph nodes at the mid-esophagus are dissected.

**Fig. 3:**
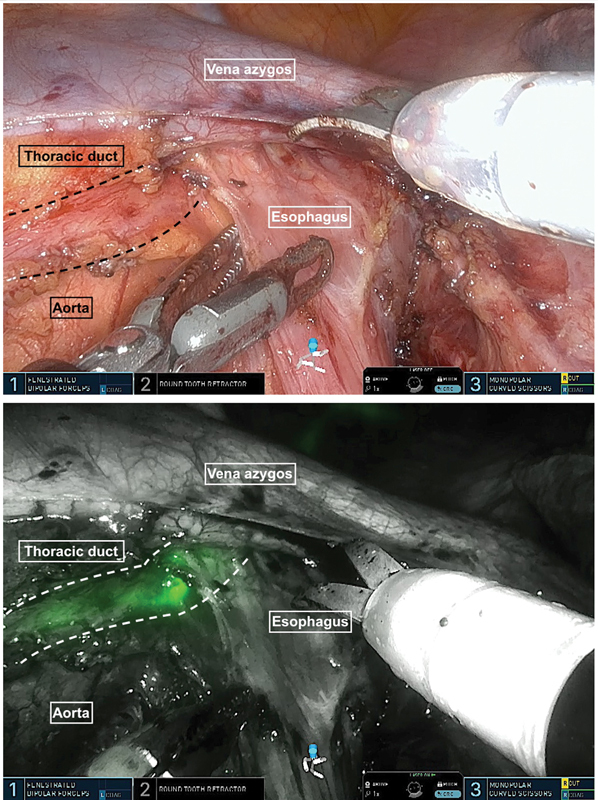
Indocyanine green (ICG) can be applied during laparoscopy so that the thoracic duct can be displayed and preserved, if indicated.

**Fig. 4:**
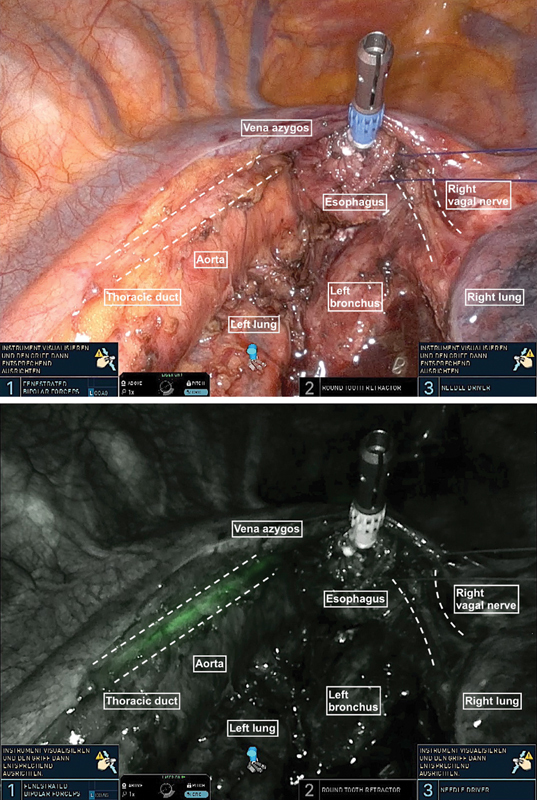
Care is taken to preserve the bronchial branches of the right vagal nerve.

**Fig. 5:**
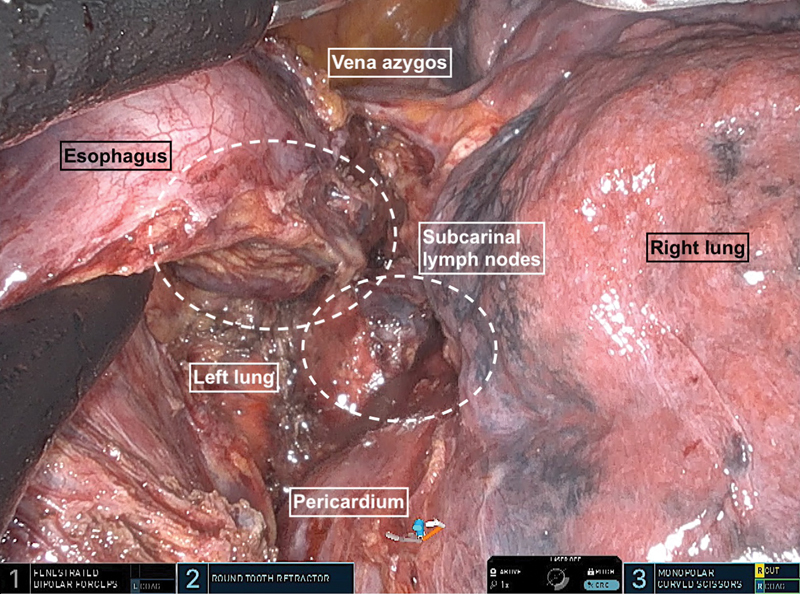
The lymph nodes at the tracheal bifurcation are completely dissected and left with the specimen.

After repositioning the patient in the left lateral to semiprone position, thoracoscopy is performed via a 12-mm optic trocar inserted through the seventh intercostal space. An additional subcostal incision approximately 4 cm wide is made at the level of the posterior axillary line, and the thoracic cavity is entered while ensuring that the diaphragm is not injured. Stay sutures are placed to fix the diaphragm to the fascia. A large SP access port (SP access Port Kit, large incision [2–7 cm]) is inserted, and a pressure of 7 mm Hg is applied to induce capnopneumothorax ( Fig. 1 ). The da Vinci SP patient-side cart is positioned caudally on the right side of the patient, and the robot is docked. The choice of instruments is at the surgeon's discretion. We used fenestrated bipolar forceps in position 1, a round tooth retractor in position 2, and scissors in position 3. A 0-degree camera is placed in a “cobra” position. After the lung has collapsed and the pulmonary ligament and pleura have been divided, the pleura is separated from the pericardium towards the right main bronchus and along the azygos vein, which can be preserved. The esophagus is tunneled underneath, and the lymph nodes at the mid-esophagus are dissected ( Fig. 2 ). The aorta can then be exposed along the azygos vein and followed to the hiatus. If indocyanine green (ICG) has been applied to the mesenteries during the abdominal phase, the thoracic duct can be displayed in *firefly mode* and can be preserved ( Fig. 3 ). The esophagus is then detached as an en bloc esophagectomy along with the mesoesophagus and freed up to the upper mediastinum. Individual bronchial arteries can be divided after they have been clipped with Hem-o-lok. At this stage, the left pleura is dissected from the pericardium, creating a bridge to the aorta and the distal esophagus, which can now be lifted with the round tooth retractor. Care is taken to preserve the bronchial branches of the right vagal nerve ( Fig. 4 ). The lymph nodes at the tracheal bifurcation are completely dissected and left with the specimen ( Fig. 5 ). This is followed by lymphadenectomy at the inferior paratracheal lymph nodes station. The skeletonized upper esophagus is then divided with the scissors, and a 2/0 Prolene purse-string suture is placed in the esophageal stump.

#### Reconstruction

After the robot is de-docked, the specimen is resected, and the anvil of the EEA 25- or 28-mm circular stapler (Covidien) is inserted into the esophageal stump through the subcostal approach. The purse-string suture is tied extracorporeally. The prepared gastric tube is luxated into the thorax in its entire length. The stapler is then introduced into the gastric tube at the appropriate site following a gastrotomy. Subsequently, an end-to-side stapled esophagogastrostomy to the posterior gastric wall is performed. The remaining portion of the stomach is resected with an Endo GIA 60 mm violet stapler cartridge, and the specimen is retrieved through the subcostal access. The robot is re-docked to create an omental flap, which is covered by the pleural tent that was previously created. After the robot is de-docked, a 20-Ch thoracic drain is inserted via the assistant trocar, and skin sutures mark the end of the procedure.

## Discussion

The SP SC RAMIE method allows for efficient total esophageal mobilization and dissection within an acceptable timeframe, along with radical mediastinal lymphadenectomy. Patients do not require paravertebral or peridural anesthesia because they do not have severe postoperative pain. The da Vinci SP surgical system and the SP SC RAMIE method have demonstrated excellent feasibility for paratracheal and subcarinal lymphadenectomy, as well as comprehensive intrathoracic esophageal mobilization. However, further investigation will confirm the feasibility, reproducibility, and clinical utility of the SP SC RAMIE. This method offers several noteworthy advantages, including controlled esophageal dissection, reduced pain, quicker patient mobilization, improved cosmetic outcomes, and improved adherence to enhanced recovery after surgery (ERAS) protocols, all attributed to the small single incision. The subcostal approach also provides easier specimen removal with wound protection and reduces postoperative pain, especially for patients with narrow intercostal spaces. 6 It can be particularly advantageous for frail patients with comorbidities.
